# Terahertz Detectors Using Microelectromechanical System Resonators

**DOI:** 10.3390/s23135938

**Published:** 2023-06-26

**Authors:** Chao Li, Ya Zhang, Kazuhiko Hirakawa

**Affiliations:** 1 Institute of Engineering, Tokyo University of Agriculture and Technology, Koganei-shi 184-8588, Japan; 2 Institute of Industrial Science, University of Tokyo, Meguro-ku 153-8505, Japan; 3 Institute for Nano Quantum Information Electronics, University of Tokyo, Meguro-ku 153-8505, Japan

**Keywords:** terahertz (THz) detection, microelectromechanical system (MEMS) resonators, bolometer, thermal sensitivity

## Abstract

The doubly clamped microelectromechanical system (MEMS) beam resonators exhibit extremely high sensitivity to tiny changes in the resonance frequency owing to their high quality (Q-) factors, even at room temperature. Such a sensitive frequency-shift scheme is very attractive for fast and highly sensitive terahertz (THz) detection. The MEMS resonator absorbs THz radiation and induces a temperature rise, leading to a shift in its resonance frequency. This frequency shift is proportional to the amount of THz radiation absorbed by the resonator and can be detected and quantified, thereby allowing the THz radiation to be measured. In this review, we present an overview of the THz bolometer based on the doubly clamped MEMS beam resonators in the aspects of working principle, readout, detection speed, sensitivity, and attempts at improving the performance. This allows one to have a comprehensive view of such a novel THz detector.

## 1. Introduction

Terahertz (THz) radiation generally refers to the spectral region that lies between the microwave and the infrared (IR) regions, spanning a frequency range of 0.1 to 10 THz [1], and possesses a dual characteristic of both microwaves and light waves. As a result of this intriguing property, THz technology has garnered significant research attention and has become a hotspot in the field of electromagnetic waves. However, going up from the well-established microwave electronics to the high-frequency THz region as well as going down from the IR photonics to the lower-photon-energy THz has been very challenging, which is called the “THz gap” [2].

Nevertheless, over the last three decades, numerous breakthroughs in THz systems and the advent of some milestone achievements (e.g., THz time-domain spectroscopy [3], THz imaging [4]) have facilitated the expansion of THz technology into various fields, such as optics [5,6], materials science [7,8,9,10], biomedical science [11,12,13], agriculture [14,15,16,17], security [18,19,20], and communication technology [21,22,23,24]. Especially given its advantages in communication transmission, THz technology is also widely considered the most promising contender for next-generation wireless communication, i.e., 6G communication [25,26]. It is noteworthy that the vast majority of research efforts in the above-mentioned applications hinge primarily on the availability of THz detectors and sources. That is why interest in THz research activities so far has been devoted to advancing the development of THz radiation generation and detection technology. One aspect limiting the exploitation of the THz band is the production of high-power sources and sensitive detectors of THz radiation. With the advent of the quantum cascade laser (QCL) [27], the field of THz technology has witnessed a transition from laboratory research to practical commercial applications. Nevertheless, the development of effective THz detection techniques is still largely impeded by the lack of reliable detectors that can operate at room temperature.

THz detectors convert THz radiation into a measurable signal through the use of materials that exhibit changes in their physical or electrical properties in response to THz radiation. In other words, a THz detector is a specific form of transducer that converts information from one form to another. There exist numerous innovative THz detectors that operate on diverse working principles, such as rectifiers, photoconductive detectors, and thermal detectors. Note that certain detectors may not fit precisely within these three categories due to the limitations of our knowledge. For a comprehensive understanding of THz detectors, readers are encouraged to consult previous review papers [28,29,30].

THz rectifiers work by converting THz electromagnetic waves into a DC signal (i.e., rectifying THz radiation) through their nonlinear current-voltage characteristics. The most common THz rectifier is the Schottky diode, introduced to THz applications by Young and Irvin in 1965 [31]. The metal, often a high-work-function metal like gold or platinum, forms an electrical contact with a semiconductor material, such as GaAs or InGaAs [32,33,34,35]. The junction between the metal and the semiconductor acts as a rectifying barrier. When THz radiation is incident on the Schottky diode, it creates an alternating electric field that induces a current. This current results in the rectification of the THz wave, converting it into a rectified DC signal. The rectification occurs due to the asymmetrical nature of the metal-semiconductor junction, which allows the flow of current in one direction while blocking it in the opposite direction. The rectified DC signal represents the envelope of the THz waveform, enabling the detection and extraction of the THz signal. Schottky diodes exhibit high sensitivity to THz radiation, especially in the low-frequency THz range (typically, noise equivalent power (NEP) ~0.5–10 pW/Hz^1/2^ [36]), and outperform other room temperature detectors in terms of response time. However, Schottky diodes operate only at low frequencies (<1.5 THz) because the operation speed of rectification is limited by the transit time of carriers as well as the RC time constants of the device structures. The recent development of Fermi-Level managed barrier (FMB) diodes [37,38,39] utilizes the low barrier height of semiconductor heterojunctions to achieve lower NEP and a broader operation bandwidth than Schottky diodes. Nevertheless, the performance and efficiency of THz rectifiers tend to degrade as the frequency increases [36], which limits their usefulness for frequencies above 1.5 THz.

In photoconductive detectors, solid-state devices composed of materials, such as superconductors and semiconductors, generally have energy level spacing that corresponds to the THz photon energy (1–100 meV) [40]. It follows that, when the solid-state device is illuminated with THz radiation, the radiation is absorbed within the material by interaction with electrons either bound to lattice atoms or to impurity atoms or with free electrons, and photoexcited carriers are generated in the device, resulting in the conversion of optical signals to electrical signals. Photoconductive detectors usually exhibit high sensitivities, but only at low temperatures due to the thermal excitation of carriers. Consequently, photoconductive detectors require cryogenic cooling to achieve optimum performance, which greatly limits their application. Moreover, photoconductive detectors exhibit wavelength selectivity because of the influence of the width of the semiconductor band gap, i.e., they only respond to THz radiation at a specific wavelength.

In contrast, thermal detectors are less sensitive than photoconductive detectors but nonselective to wavelength, and some of them can be operated at room temperature [28,41]. On the other hand, the manufacturing cost and the operation difficulty of thermal detectors are reduced compared with photoconductive detectors, which is why thermal detectors are preferred for commercial applications. A typical thermal detector includes an absorber of THz radiation with heat capacitance, *C*_th_, and a heat sink (thermal reservoir) with temperature, *T_s_*, connected to the absorber by a thermal link with thermal conductance, *G*_th_. The incident radiation is absorbed to change the detector temperature, and the resultant changes in certain physical properties are finally output as detectable electrical signals. Representative devices of this type for THz detection include Golay cells, pyroelectric detectors, and bolometers [36].

The Golay cell consists of an enclosed chamber filled with argon or xenon, a radiation absorber, and a flexible membrane with a mirror attached. The thermal expansion of the gas due to THz radiation causes the flexible membrane to deform. Such a deformation deflects a beam of light shining on a photocell, thereby producing a change in the photocell current as the output [42,43]. Golay cells exhibit good sensitivity (10^5^–10^6^ V/W) at room temperature but have a relatively long response time (typically, 15 ms). Other drawbacks, such as difficulty in assembling large arrays and sensitivity to mechanical vibrations, also limit the application potential of the Golay cell [44].

Pyroelectric detectors are typically fabricated from ferroelectric (pyroelectric) crystals that exhibit strong temperature-dependent electric polarization [45,46,47]. Temperature fluctuations induced by THz radiation lead to charge polarization on the surface of pyroelectric crystals. This results in the formation of a potential difference at both ends of the crystal, which can be utilized to estimate the power of the THz radiation. However, since the potential difference eventually fades away with constant radiation power, THz radiation needs to be modulated for detection by pyroelectric detectors. In comparison to Golay cells, pyroelectric detectors exhibit a lower responsivity (typically 5–300 kV/W [36]) and a longer response time (typically 10–50 ms [36]), as well as large internal thermal noise. However, it has the advantage of being compact, robust, and not sensitive to mechanical vibration.

A bolometer typically consists of an absorber, a thermistor, and a thermal link. Its working principle is similar to that of Golay cells and pyroelectric detectors, but the output signal is based on the change in resistance of the temperature-dependent thermistor [48,49,50]. Traditional bolometers composed of metal thermistors have long been treated as slow and low-sensitivity devices. With the application of semiconductor and superconductor technologies to the fabrication of bolometers, relatively fast and highly sensitive detections have been realized [51,52,53]. Nevertheless, those semiconductor or superconductor-based bolometers are often cooled down to liquid helium temperature to achieve high thermistor sensitivities. Cumbersome cooling equipment limits the path to miniaturization and integration of devices. Microbolometers made of vanadium oxide (VOx) have been reported that can work as sensitive THz detectors for THz imaging and spectroscopic applications at room temperature [54,55,56]. However, due to the limitation of still relying on resistance change as the working principle, the response time of such bolometers is not encouraging. Therefore, as long as the principle of detecting THz waves through resistance changes in semiconductors and superconductors is used, achieving highly sensitive detectors at room temperature remains extremely challenging. To overcome this limitation, alternative physical quantities for THz detection need to be considered.

The use of micro/nano-electromechanical system (MEMS/NEMS) resonators for THz detection has been considered a promising approach owing to their potential for achieving high sensitivity, fast response, compactness, and miniaturization [57,58,59]. Furthermore, MEMS/NEMS resonators can be fabricated using microfabrication techniques, which makes them relatively inexpensive and easy to mass-produce. They also have the potential for integration with other MEMS devices, such as filters, switches, and amplifiers, for on-chip signal processing [60,61]. The MEMS resonator, typically a microcantilever or a doubly-clamped beam, can be driven into resonant oscillation by an external circuit and work as a bolometer for THz radiation detection. When the MEMS resonator absorbs THz radiation, a temperature rise in the resonator will be induced, which changes its mechanical properties and leads to a shift in its resonance frequency. This frequency shift is proportional to the power of THz radiation absorbed by the resonator and can be detected and quantified, thereby allowing the THz radiation to be measured. Owing to the high quality factor (Q-factor) of MEMS resonators (thousands to ten thousand or even more at room temperature), which is a measure of the sharpness of the resonance peak, they exhibit very high sensitivity to small changes in resonance frequency, thus allowing for highly sensitive THz detection. Moreover, since the thermal capacity of MEMS resonators is tiny, high-speed THz detection is achievable.

Overall, MEMS/NEMS resonators offer advantages in fast and highly sensitive THz detection, which is also the core of this review. It should be noted that in what follows, we will refer to the THz detection element using MEMS as a “MEMS bolometer” in some places.

In this review, we start with a concise introduction to definitions of what is meant by THz radiation and by THz detectors and sketch the characteristics of such detectors. While the topic of THz detectors has been reviewed numerous times previously [28,29,30], the emphasis is on novel THz detectors using MEMS/NEMS resonators in this work. This is followed by a concise survey of existing THz/IR detectors based on MEM/NEMS resonators. Next, based on our own work, we give a comprehensive introduction to THz bolometers using doubly clamped MEMS beam resonators based on GaAs in Section 3, and Section 4 deals with attempts to improve the performance of MEMS bolometers. The last section sums up the field as a whole and puts forward the prospects and remaining challenges of THz detectors based on MEMS/NEMS resonators.

## 2. THz/IR Detection Based on MEMS/NEMS Resonators

MEMS/NEMS resonators possess an exceptionally high sensitivity to tiny changes in certain physical quantities (e.g., resonance frequency, oscillation amplitude, or displacement), owing to their high Q-factors. This sensitivity characteristic is very attractive for sensing applications and has been utilized for the detection of mass [62,63,64], spin orientation [65,66], charge [67,68,69], and temperature [70,71]. In more recent years, the sensing applications of MEMS/NEMS resonators have been expanded to include THz/IR detection. Given that the radiation power of THz/IR is ultra-small, detectors for these frequencies require very high sensitivity with respect to temperature. A number of groups have demonstrated that MEMS/NEMS resonators can work as fast and highly sensitive THz/IR detectors. Depending on the vibrational mode and geometrical shape, they can be categorized as torsional mode [72,73,74,75,76], bending mode [59,77], or membranous shape [57,78,79]. Table 1 provides a survey of the performance of THz/IR detectors based on MEMS/NEMS resonators, allowing for an objective comparison of existing MEMS bolometers.

**Figure 1:**
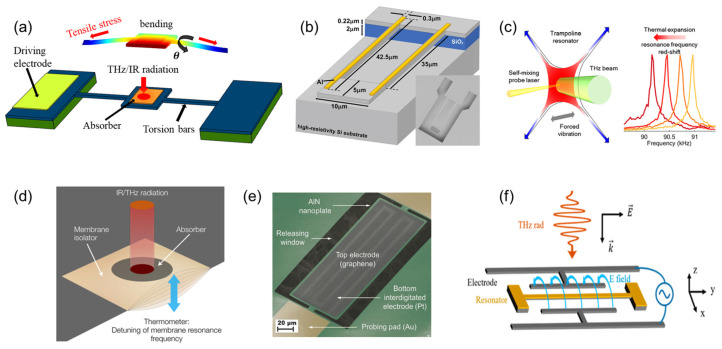
(**a**) Schematic illustration of a typical resonator based on torsional mode. (**b**) Schematic of the cantilever geometry with all relevant dimensions. The Al antennas are 80 nm thick. The inset shows a SEM image of the fabricated device. Reprinted from [77], with the permission of AIP Publishing. (**c**) Schematic illustration of a trampoline membrane resonator and its working principle. Reprinted from [57], with the permission of Vicarelli et al. (**d**) Schematic drawing of the silicon nitride drum resonator. The detector is based on a 50-nanometer-thick drum resonator made of silicon nitride, comprising an absorber layer. The absorber’s thin film thermalizes part of the radiation. The resulting photothermal heating of the nanoelectromechanical drum causes a measurable detuning of the drum’s resonance frequency. Reprinted from [79] with the permission of Piller et al. (**e**) A false-colored tilted SEM image of a fabricated G-AlN NEMS resonant IR detector. The device is 75 μm wide and 200 μm long. Reprinted with permission from [78]. Copyright 2016 Qian et al. (**f**) Schematic of the optomechanical system, with indications of the RF voltage and THz radiation. Reprinted with permission from [58]. Copyright 2022 American Chemical Society.

Resonators based on torsional mode, as typically shown in Figure 1a, are composed of torsion bars (torsional springs) and a resonator body (film or plate) with an absorber deposited on it. When an incident electromagnetic wave is absorbed, the resonator body bends upward due to the larger thermal expansion of the layered structure at the center. This upward bending makes the torsion bar slant against the line connecting the fixed ends and generates a hard spring effect in the torsion bars that support the resonator body, thereby increasing its resonant frequency. The resulting frequency change is utilized to estimate the sensitivity of the resonators with respect to the radiation power. Although these resonators share similar structures, their performance differs based on their dimensions, fabrication process, and materials. Laurent et al. [72] demonstrate a 12-micrometer-pitch nanoelectromechanical resonant IR sensor with fully integrated capacitive transduction. A low-temperature fabrication process is used to manufacture torsional resonator arrays. At room temperature, this sensor shows a NEP of 27 pW/Hz^1/2^ and a NETD of 2 K for a 50-Hz noise bandwidth. Yamazaki et al. proposed a torsional resonator made of a tense thin film of polycrystalline Si [76], which exhibits a sensitivity of 30 Hz/K and a thermal coefficient of resonant frequency (TCF) of 830 ppm/K for IR detection. In their later work [75], this TCF is increased to 1000 ppm/K by using the metal-induced lateral crystallization of a hydrogenated amorphous Si (a-Si:H) thin film to form a tension-enhanced poly-Si film. Gokhale et al. [74], for the first time, demonstrate the use of gallium nitride (GaN)-based micromechanical resonator arrays as high-sensitivity, low-noise IR detectors, in which each individual pixel in the array is a thin-film resonator mechanically suspended by thin tethers. For a 100 mK radiation-induced temperature rise, it exhibits a radiant responsivity of 0.0168 W^−1^ and a thermal time constant on the order of 556 µs. Zhang et al. [73] develop an IR bolometer based on a micrometer-sized torsional resonator with nanoscale supporting rods (~1 μm long and 50–100 nm in diameter). This nanoscale design provides both extraordinary thermal isolation and excellent torque sensitivities, resulting in a NETD of 390 mK. Moreover, they also mentioned that a room-temperature NETD below 10 mK appears feasible with further scaling and optimization.

To date and to our knowledge, only one work [77] has demonstrated a MEMS resonator based on bending mode for THz detection, in addition to the previously reported GaAs double-clamped MEMS beam bolometer [59]. In that work, as schematically shown in Figure 1b, the resonator is fabricated as a suspended U-shaped cantilever of micrometric size, on top of which two aluminum half-wave dipole antennas are deposited. Unlike the GaAs double-clamped MEMS beam bolometer [59], the responsivity of the cantilever resonator is estimated by the out-of-plane displacement induced by the THz radiation rather than the frequency shift. This cantilever resonator exhibits a responsivity with respect to displacement of 1.5 × 10^8^ pm W^−1^, corresponding to a NEP of 20 nW/Hz^1/2^ at 2.5 THz, as well as a thermal response time of 2.5 µs.

Regarding the resonators based on membranous shape, an Italian research group proposes a trampoline membrane structure based on silicon nitride [57], as schematically shown in Figure 1c. The absorbed THz radiation heats up and thermally tensions the suspended membrane, thereby shifting its resonance frequency. In this work, the MEMS bolometer shows a minimum NEP of ~100 pW/Hz^1/2^ and a detection speed of 40 Hz at room temperature, challenging the state-of-the-art bolometric detectors in the sub-THz range (140 GHz). Furthermore, Sadeghi et al. [83] demonstrate that further improvement in thermal responsivity can be achieved by embedding a phononic crystal membrane into a nanomechanical trampoline. Furthermore, based on silicon nitride, Piller et al. [79] propose a drum NEMS resonator for IR detection, as shown in Figure 1d, which is made of 50 nanometer-thick low-stress silicon comprising an absorber layer. Its working principle is also based on the thermally induced detuning of the resonance frequency. With an IR range of 5 to 20 µm, the drum NEMS resonator achieves a thermal time constant of 17 ms and a NEP of 320 pW/Hz^1/2^. It is worth noting that this NEP corresponds to the situation where the IR spot is fully filling the drum area. However, a smaller NEP of 30 pW/Hz^1/2^ is feasible by optimizing the IR spot size. The electromechanical and thermal properties of the resonator might be deteriorated by the relatively bulky IR-absorbing material (or material stack) attached to the resonator body. To address this issue, a monolayer graphene sheet is utilized in lieu of a conventional metal film to fabricate the IR bolometer [78], as shown in Figure 1e, which is a thin graphene-aluminum nitride plate. Through this, the Q-factor and IR absorptance are improved by 12.6% and a factor of 10 (at 3.4 µm wavelength) and 138 (at 5 µm wavelength), respectively, compared with their metal-electrode counterparts. This results in a thermal time constant of ~0.53 ms and a NEP of ~47 nW/Hz^1/2^ for the presented IR bolometer, which is a promising candidate for ultrafast and high-resolution NEMS IR detectors.

Furthermore, there have been reports on MEMS/NEMS resonators based on optical readout for THz detection [58,81,82], which employ combined Coulomb/photothermal detection. Here, we present an example of this type of resonator. Figure 1f shows the schematic of the optomechanical system, in which the resonator is designed in a dog-bone shape [58]. Its optical readout setup is discussed in Ref. [81]. The excitation of the resonator by the radiation source induces eddy currents, which heat up the mechanical nanobeam and generate thermal strain, leading to light modulation and revealing the change in its resonance frequency, vibration amplitude, and phase. With a 2.5-THz QCL radiation source, the resonator shows a thermal time constant of 1.2 μs and a responsivity of 30 pm/nW. Furthermore, in this optomechanical system, THz-induced Duffing nonlinearity is observed, which opens a new possibility for THz sensing, possibly taking advantage of nonlinear interactions.

Overall, reports on MEMS resonators for THz detection are significantly fewer than those for IR detection. THz detection is more challenging than IR detection, requiring higher sensitivity, a better signal-to-noise ratio, specific materials, and thermal noise control. However, with advancements in material science and device manufacturing technology, THz detection technology has made significant progress, offering broad prospects for THz technology applications.

## 3. THz Bolometer Using GaAs Doubly Clamped MEMS Beam Resonators

In this section, we provide more elaboration on the THz bolometer using GaAs doubly clamped MEMS beam resonators, including the working principle, responsivity, and noise performance. In what follows, “MEMS bolometer” refers only to the “GaAs beam bolometer”.

### 3.1. Working Principle and Fabrication of GaAs MEMS Bolometer

**Figure 2:**
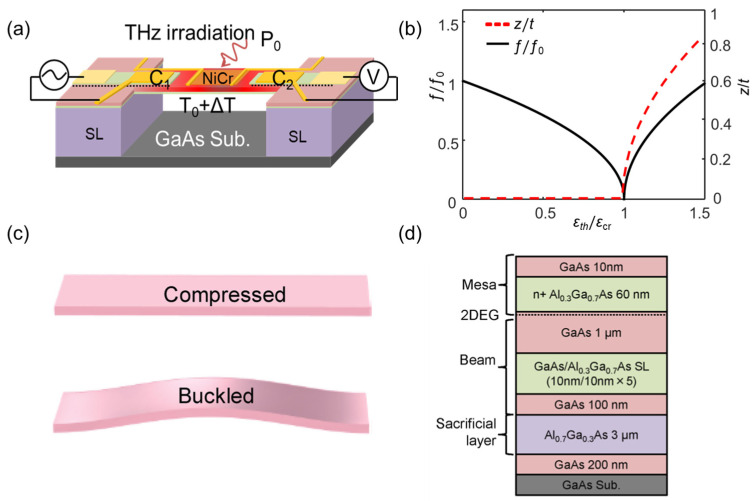
(**a**) Schematic illustration of the doubly clamped MEMS beam resonator. The conduction layer and the top gates on both ends of the beam form two piezoelectric capacitors, C_1_ and C_2_. A thin NiCr layer is deposited as a THz absorber and is used for calibrating the responsivity of the MEMS beam resonators. (**b**) The calculated resonance frequency (ƒ/ƒ_0_) as a function of *ε*_th_/*ε*_cr_ for various beam center deflections (*z*/*t*), the ƒ, *ε*_th_, and *z* are normalized by the natural frequencies, ƒ_0_, *ε*_th_, and beam thickness, *t*, respectively. (**c**) Schematic illustrations of the beam structures that experience compressive stress (**top**) and buckling (**bottom**). Reprinted from [88], with the permission of AIP Publishing. (**d**) Wafer structure used to fabricate the doubly clamped GaAs beam resonator. Reprinted from [59], with the permission of AIP Publishing.

The GaAs doubly clamped MEMS bolometer is schematically shown in Figure 2a, which works by sensitively reading the THz-induced temperature rise in the MEMS beam as a shift in the resonance frequency of MEMS resonators. The eigen-frequency of a doubly clamped MEMS beam for the *n*th vibrational mode is given by [84]:

(1) $$f_{n}=\frac{\omega_{n}}{2\pi }=\frac{t}{L^{2}}\sqrt{\frac{E}{12\rho }}\lambda_{n}^{2},$$

where *ω_n_* is the angular frequency of the *n*th vibrational mode, and *t*, *L,* and *ρ* represent the thickness, length, and density of the MEMS beam, respectively. *E* is the Young’s modulus of material. *λ_n_* is the nondimensional eigenvalue for the *n*th vibrational mode; the first few values are:

(2) $$\lambda_{1}=4.73,\;\;\lambda_{2}=7.853,\;\;\lambda_{3}=10.996,\;\;\lambda_{4}=14.1372,\ldots$$

Usually, the first bending mode of the MEMS beam is employed, as its resonance frequency change is affected by the thermal strain (*ε*_th_) the most. The resonance frequency of MEMS bolometers related to such thermal strain is given as [85]:

(3) $$f=f_{0}\sqrt{1-\frac{\varepsilon_{\mathrm{th}}}{\varepsilon_{\mathrm{cr}}}+\frac{3z^{2}}{2t^{2}}},$$

where *f*_0_ is the eigen-frequency (natural) frequency of the first bending mode. *z* is the beam center deflection. Here, *ε*_cr_ is Euler’s critical buckling strain of the MEMS beam, which can be calculated by using the model developed in Refs. [86,87] as:

(4) $$\varepsilon_{\mathrm{cr}}=\frac{\pi^{2}}{3}\frac{t^{2}}{L^{2}}$$

The resonance frequency, *f*, depends on not only *ε*_th_ but also *z*. Figure 2b shows the normalized resonance frequency (ƒ/ƒ_0_) and beam center deflection (*z*/*t*) as a function of *ε*_th_/*ε*_cr_. For an ideal, flat MEMS beam without center deflection (*z* = 0), as indicated by the top of Figure 2c, when *ε*_th_/*ε*_cr_ < 1, the MEMS beam remains flat (*z* = 0), and *f* decreases with *ε*_th_ and drops to zero at *ε*_th_/*ε*_cr_ = 1. However, when *ε*_th_ exceeds *ε*_cr_, the beam shows a deflection (*z* > 0) caused by buckling, as indicated by the bottom of Figure 2c, and the resonance frequency starts increasing with a further increase in *ε*_th_. It should be noted that the strain term remains *ε*_th_/*ε*_cr_ = 1 after the buckling of the MEMS beam, which is because the excess *ε*_th_ is translated to the increased beam deflection. In other words, the resonance frequency is only determined by *z* after the critical buckling condition, which can be expressed as:

(5) $$z=\frac{2L}{\pi }\sqrt{\left(\varepsilon_{\mathrm{th}}-\varepsilon_{\mathrm{cr}}\right)},$$

When THz radiation with power *P*_0_ is irradiated on the beam, the beam expands thermally, and compressive thermal strain, *ε*_th_, develops in the beam owing to the doubly-clamped structure, which causes the change in resonance frequency of the MEMS bolometer as shown in Figure 2b. Assuming the initial strain of the MEMS beam is zero, the thermal responsivity of MEMS bolometers, *R*, can be defined as the change in resonance frequency (Δ*f*) with respect to unit incident power, which is expressed as [85]:

(6) $$R\equiv \frac{\Delta f}{f_{0}}\frac{1}{P_{0}}=\frac{3}{4\lambda_{1}^{2}}\eta \frac{\alpha_{T}}{\kappa }\frac{1}{w}{\left(\frac{L}{t}\right)}^{3},$$

where *η* is the THz absorptance of the absorber, *α*_T_ is the thermal expansion coefficient, *κ* is the thermal conductivity of the beam material, and *w* is the width of the beam. As we can see from Equation (6), the responsivity of a MEMS bolometer is proportional to the frequency shift with respect to unit incident power, i.e., the slope of the black curve in Figure 2b. Since the slope changes with the applied strain, the *R* is usually estimated by linear fitting within a small heating power range. From the perspective of dimensions, the responsivity is proportional to (*L*/*t*)^3^, so the sensitivity can be greatly increased by increasing the aspect ratio of the MEMS beam. However, a too large aspect ratio will make the MEMS beam very easily affected by the initial internal strain [85]. From the perspective of physical properties, the responsivity shown in Equation (6) is determined by the thermal expansion coefficient and thermal conductivity of the beam material in addition to the THz absorptance and the beam dimensions, thus allowing for good reproducibility of responsivity that does not rely on fabrication details of the device. Some feasible approaches to improving the responsiveness of MEMS bolometers will be discussed in detail in Section 4.

Figure 2d shows the wafer structure used for fabricating the doubly clamped GaAs beam resonator. After growing a 200-nanometer-thick GaAs buffer layer and a 3-micrometer-thick Al_0.7_Ga_0.3_As sacrificial layer on a (100)-oriented semi-insulating GaAs substrate, the beam layer is formed by depositing a 100-nanometer-thick GaAs layer, a GaAs/Al_0.3_Ga_0.7_As superlattice structure, and a 1-micrometer-thick GaAs layer. Subsequently, a two-dimensional electron gas (2DEG) is formed by growing a 60-nanometer-thick Si-doped Al_0.3_Ga_0.7_As layer and a 10-nanometer-thick GaAs capping layer. The suspended beam structure is formed by selectively etching the sacrificial layer with diluted hydrofluoric acid (HF), as schematically shown in Figure 2a. The 2DEG layer and the top gate electrodes on the two ends of the beam form two piezoelectric capacitors, C_1_ and C_2_. Utilizing the piezoelectric effect of GaAs developed by NTT Basic Research Laboratories [89], an AC voltage is applied to C_1_ to drive the beam, and the induced oscillation signal is detected by C_2_. In order to obtain a broad sensitivity spectrum independent of wavelength, an impedance-matched NiCr layer is deposited on the MEMS beam as a THz absorber.

Figure 2d shows the normalized frequency shift as a function of heating power, *P*_in_, where *P*_in_ is generated by applying a DC voltage to the NiCr film in order to simulate heating by THz radiation. As seen in the figure, when the heating power is increased, the resonance frequency is reduced due to the thermal strain. The frequency shift keeps good linearity up to the mW-range, and the responsivity is estimated as ~79.5 W^−1^. This is typical for a MEMS beam with a geometry of 100 × 30 × 1.2 μm^3^. The Q-factor of the MEMS resonator is typically ~6000 for these GaAs MEMS resonators. at room temperature.

### 3.2. Signal Readout and Operating Speed of MEMS Bolometer

**Figure 3:**
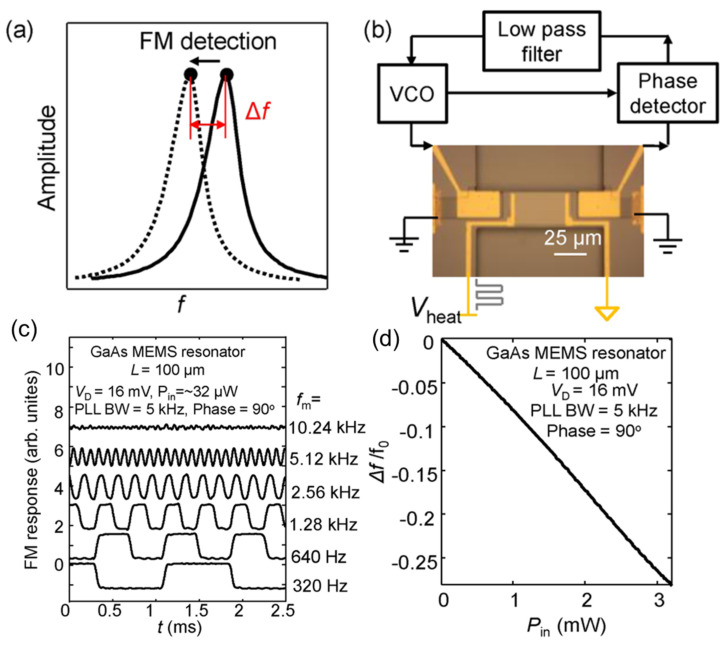
(**a**) Schematic diagram of the FM detection. Reprinted from [59], with the permission of AIP Publishing. (**b**) Circuit illustration of the FM detection. The MEMS beam resonator achieves a self-sustained oscillation mode with a PLL. The modulated voltages with various modulation frequencies *f*_m_ are applied to the NiCr heater. Reprinted from [59], with the permission of AIP Publishing. (**c**) The Signal waveforms in the case of being switched on and off at *f*_m_ = 320–10,240 Hz, with the initial conditions: *V*_D_ = 16 mV, *P*_in_ = 32 µW, and the demodulation bandwidth of the PLL is 5 kHz. Reprinted from [59], with the permission of AIP Publishing. (**d**) The normalized measured frequency shift as a function of *P*_in_, using the FM detection mode. Reprinted from [59], with the permission of AIP Publishing.

Different from other thermal sensors that measure the electrical properties of the sensing material, the MEMS resonators detect the frequency shift as the output signal. Here, a frequency-modulation (FM) method with a phase-locked loop (PLL) is utilized to maintain the self-oscillation of the MEMS resonator and detect its frequency shift, as schematically shown in Figure 3a,b. It has been demonstrated that the bandwidth (BW), limited by the Q-factor of the cantilever, can be broken in the case of FM detection [90]. A key element in the detection system is the PLL, which imparts a controlled phase delay to the feedback-driving voltage in relation to the detected oscillation motion. This feedback scheme effectively addresses the damping effects present in the MEMS beam, thereby ensuring the self-sustained oscillation of the MEMS resonator [91]. Furthermore, the MEMS bolometer incorporates a built-in frequency demodulator within the PLL, enabling swift measurement of the resonance frequency shift induced by an external heat input.

The initial driving conditions for the device that demonstrate the speed of the FM detection operation are a driving voltage of *V*_D_ = 16 mV, a 90° phase delay, and a 5 kHz demodulation bandwidth of the PLL. Figure 3c plots the output signal waveforms from the PLL under the conditions switched on and off at various *f*_m_ (320–10,240 Hz) and an input heating power of *P*_in_ = 32 μW. As seen, the signal amplitude displays a gradual decrease as *f*_m_ approaches 5 kHz. The detection speed in the FM detection scheme is governed by two limiting factors. Firstly, the thermal time constant (*τ*_th_) of the system, represented by the ratio of heat capacitance (*C*_th_) to thermal conductance (*G*_th_) of the MEMS beam, significantly impacts the operational speed. The demodulation BW of the PLL circuit also plays a pivotal role in the detection speed of the system since it determines the ability of the PLL to track the frequency shift efficiently. A *τ*_th_ of ~55 μs is estimated by using the finite element method calculation, corresponding to a −3 dB bandwidth of approximately 1/2 π*τ*_th_ ≈ 2.9 kHz. Interestingly, this estimation closely aligns with the measured −3 dB bandwidth of the present MEMS resonator, which is approximately 2.5 kHz. The MEMS bolometer deserves emphasis for its remarkable speed, surpassing that of conventional room-temperature THz thermal sensors. In comparison to the widely used pyroelectric detector [92,93] and the bolometers based on the phase transition in vanadium oxide (VOx) [94,95,96], the present MEMS bolometer shows a comparable responsivity while outperforming these conventional thermal sensors in terms of operation speed by more than 100 times. This outstanding performance underscores the promising potential of the present MEMS bolometer for fast THz imaging [97] and establishes it as a compelling candidate in the field.

### 3.3. Sensitivity and Dynamical Range of MEMS Bolometer

**Figure 4:**
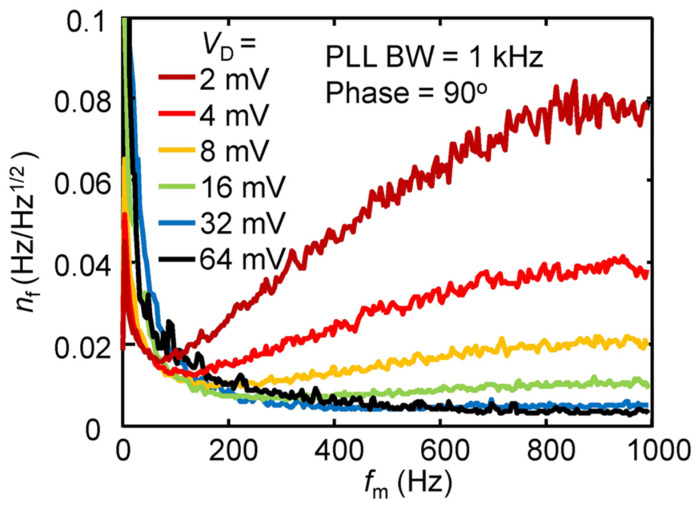
The measured frequency noise, *n*_f_, as a function of the heat modulation frequency, *f*_m_, at various driving voltages (*V*_D_ = 2, 4, 8, 16, 32, and 64 mV). The demodulation bandwidth of the PLL is set to be 1 kHz. Reprinted from [59], with the permission of AIP Publishing.

NEP is used to characterize the sensitivity of MEMS bolometers, which is defined as the power that generates a signal output that is equal to the root-mean-square (RMS) noise output in a 1 Hz bandwidth [98]. In terms of responsiveness in FM detection, the NEP is as

(7) $$\mathrm{NEP}=\frac{n_{f}}{f_{0}}\frac{1}{R},$$

where *n*_f_ represents the frequency noise spectrum. Figure 4 shows the frequency noise spectra as a function of *f*_m_ at various driving voltages. As seen, in the case of *V*_D_ ≤ 8 mV, the *n*_f_ roughly increases with the *f*_m_ at a range of *f*_m_ > 100 Hz, but the noise level is greatly reduced in the case of large driving voltages. In the FM detection mode, the *n*_f_ is widely recognized to primarily come from two sources: thermal Brownian motion [99] and residual electrical noise generated by the buffer amplifier incorporated in the measurement circuit. The thermal Brownian motion contributes to white spectral noise, while the residual electrical noise introduces frequency noise that escalates proportionally with the *f*_m_. It is worth noting that both the two noise components exhibit an inverse relationship with the oscillation amplitude of the resonator. That is why the *n*_f_ can be efficiently suppressed at a large driving voltage, as indicated by Figure 4. Consequently, a large and linear oscillation amplitude represents an effective approach to enhancing the performance of MEMS bolometers. The minimum *n*_f_ obtained in Figure 4 is 3.5 mHz/Hz^1/2^ at *f*_m_ = 1 kHz in the case of *V*_D_ = 64 mV. Furthermore, the NEP of the present MEMS bolometer can be estimated as ~90 pW/Hz^1/2^. This NEP value is comparable to that of the conventional pyroelectric and VOx sensors; however, the thermal response speed is more than 100 times faster. Furthermore, the NEP limited by the thermal fluctuation noise can be expressed as [100]:

(8) $${\mathrm{NEP}}_{\mathrm{TF}}={\left(4k_{B}T^{2}G_{\mathrm{th}}\right)}^{\frac{1}{2}},$$

where *k*_B_ is the Boltzmann constant and *T* is the temperature. The typical NEP_TF_ is 20 pW/Hz^1/2^, which is in the same order of magnitude as the measured NEP, indicating that the sensitivity of the present MEMS bolometer is already close to the theoretical limit due to the thermal fluctuation noise [100,101].

In addition to response speed, the present MEMS bolometer also outperforms in its dynamic range. In contrast to other sensitive THz bolometers that have limited capacity for handling small heat powers, the present MEMS bolometer exhibits the ability to accommodate significantly larger power inputs. For instance, the VOx bolometers undergo a phase transition over a temperature range of 10 K [96], resulting in a maximum estimated input power of Δ*T* × *G*_th_ *≈* 1 μW, considering the small *G*_th_ (~0.1 μW/K) [94]. By contrast, the presented MEMS bolometer works by frequency detection, maintaining good linearity across a wide heat range of 0–3 mW, as shown in Figure 3d. This range is approximately 3000 times larger than that achievable with VOx bolometers. In the case of a 1 Hz detection BW and an acceptable signal-to-noise ratio (SNR = 1), the minimum detectable power, *P*_min_, can be estimated as NEP × BW^0.5^ × SNR = 90 pW, which gives a dynamic range of over 3 × 10^7^.

### 3.4. Optical Characterization

**Figure 5:**
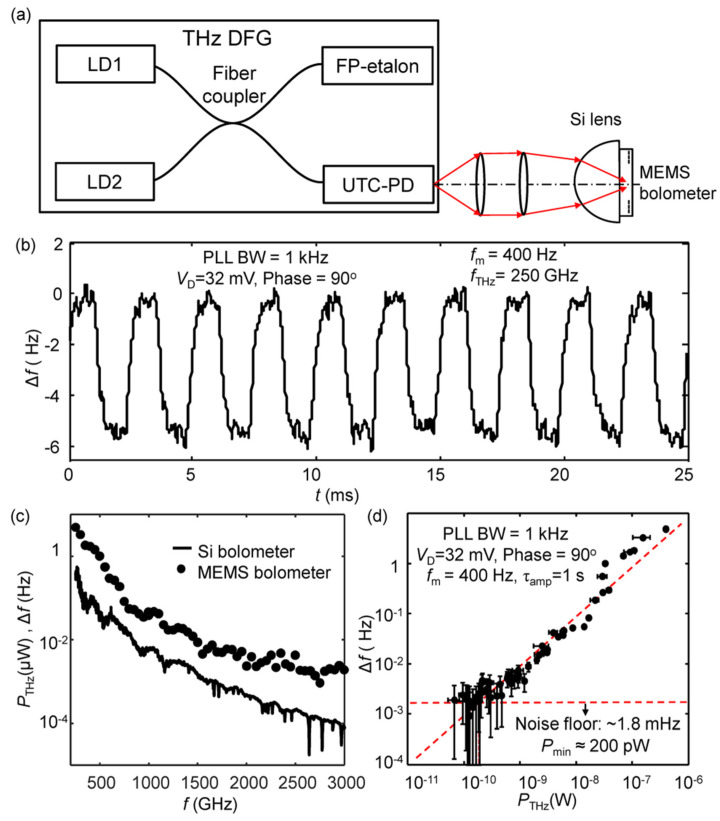
(**a**) A schematic diagram for the optical measurements that use a monochromatic THz DFG source. LD1 and LD2 are two tunable lasers that have a lasting frequency difference in the THz range. A uni-traveling-carrier photodiode (UTC-PD) is used to generate THz radiation by using the mixed output of LD1 and LD2. The MEMS resonator is mounted on a silicon hyper-hemispherical lens to focus the incident THz radiation on the MEMS beam. Reprinted from [59], with the permission of AIP Publishing. (**b**) The detected frequency shift of the MEMS bolometer when the output THz power of the DFG source is switched on and off. The output frequency of the THz DFG, *f*_THz_, is set to be 250 GHz. The THz radiation is modulated at 400 Hz. The output power, *P*_THz_, is 0.44 μW. The MEMS resonator was operated in FM detection mode with a PLL demodulation bandwidth of 1 kHz. Reprinted from [59], with the permission of AIP Publishing. (**c**) THz power measured by a Si composite bolometer as the THz DFG frequency is scanned from 250 GHz to 3.0 THz. The THz output power decreases from 0.44 μW to 80 pW as the DFG frequency increases. The dots show the measured frequency shift, Δ*f*, of the MEMS bolometer. Δ*f* is measured by using a lock-in time constant, *τ*_amp_, of 1 s at 300 K. Reprinted from [59], with the permission of AIP Publishing. (**d**) Output signal of the MEMS bolometer, Δ*f*, as a function of *P*_THz_. The noise floor of the present MEMS bolometer is ~1.8 mHz. Using this value, the minimum detectable power, *P*_min_, is determined to be ~200 pW. Reprinted from [59], with the permission of AIP Publishing.

The optical performance of the present MEMS bolometer is examined by using a continuous-wave monochromatic, single-mode THz light source employing difference frequency generation (DFG) [102]. The experimental setup depicted in Figure 5a involves the utilization of a silicon hyper-hemispherical lens to focus the THz radiation onto the MEMS beam. Operating in FM detection mode, the MEMS bolometer features a PLL demodulation BW of 1 kHz, and the modulated THz radiation is 400 Hz. The resulting frequency shift (Δ*f*) of the MEMS bolometer is measured by a lock-in amplifier with an output time constant (*τ_amp_*) of 1 s at 300 K. The output frequency (*f*_THz_) of the THz DFG is set to 250 GHz. Figure 5b shows a trace of Δ*f* when a THz radiation of 0.44 μW is switched on and off, revealing a clear and periodic frequency shift. Since the output power of the THz DFG decreases significantly with increasing *f*_THz_ [103]_,_ a wide range is achieved by sweeping *f*_THz_ from 250 GHz to 3 THz. In Figure 5c, the solid and dotted curves represent, respectively, the measured output THz power using a cryogenic Si composite bolometer and the MEMS bolometer as a function of the DFG frequency. Furthermore, Figure 5d plots the measured Δ*f* as a function of the calibrated output power of the THz DFG source, determined with a cryogenic Si composite bolometer. As seen, Δ*f* demonstrates a good linear response over a wide range of power levels. The optical *P*_min_ of the MEMS bolometer, estimated from Figure 5d, is about 200 pW. Moreover, the optical NEP can be calculated as:

(9) $$\mathrm{NEP}=\frac{P_{\min}}{\mathrm{SNR}\sqrt{1/2\pi \tau_{amp}}}\approx 500\;\mathrm{pW}/{\mathrm{Hz}}^{1/2}.$$

This optical NEP enables the measurement of THz radiation from a standard blackbody light source, as demonstrated in the supplementary material of Ref. [59]. This highlights the potential of the presented MEMS bolometer in various THz spectroscopy applications. However, the optical NEP is approximately 5.6 times larger compared to the electrical NEP (~90 pW/Hz^1/2^). This discrepancy primarily arises from the relatively small absorption coefficient of the NiCr THz absorber (~20%; calculated for the total power incident on the Si hyper-hemispherical lens) and potential losses in THz radiation collection. Enhancing the absorption coefficient can be achieved through the utilization of metamaterials [104,105] or a proper choice of substrate thickness [106].

In conclusion, THz bolometers using MEMS resonators have been shown to have high sensitivity and a fast response time, and they have potential applications in a variety of fields. However, there is still much room for improvement in the performance of MEMS bolometers. In the next section, we would like to discuss some attempts to improve the performance of MEMS bolometers.

## 4. Attempts at Improving the Performance of GaAs MEMS Bolometers

This section focuses on reviewing the attempts to improve the performance of MEMS bolometers, such as the strain tuning effect (for responsivity) [85,88,107], the control of mechanical nonlinearity (reduction of noise) [108,109], the internal mode coupling effect (for responsivity) [110], the nanometer-scale hole array structure (for response time and responsivity) [111,112], and wafer bonding (for sensitivity spectrum) [113]. In what follows, “MEMS bolometer” refers only to the “GaAs beam bolometer”, i.e., all the attempts are based on the GaAs beam bolometer we have discussed in Section 3.

### 4.1. Strain Tuning Effect on Responsivity

The thermal response of MEMS bolometers is determined by the frequency shift with respect to the thermal strain. As seen from Equation (3), the frequency-strain relation has a nonlinear form, which is also presented in Figure 2b. Thus, it is possible to improve the thermal responsivity of the MEMS resonator by modulating the internal strain [88,107]. It should be noted that the slope is dramatically enhanced near the critical buckling point (*ε*_th_/*ε*_cr_ = 1), as shown by the black curve of Figure 2b, suggesting that the responsivity becomes very large. Such a transition point is preferable for high-sensitivity sensing applications. Therefore, if a precise compressive strain is preloaded to induce the buckling of the MEMS beam, then the thermal responsivity of the MEMS beam can be greatly improved.

The use of lattice mismatch between the GaAs and InAs has been demonstrated as an effective approach to induce compressive strain in the MEMS beam [85], which is realized by adding a small amount of indium into the GaAs beam structure during the wafer growth. The lattice mismatch strain in the MEMS beam can be calculated as [85]:

(10) $$\varepsilon_{l}=\left(\frac{a_{\mathrm{InAs}}}{a_{\mathrm{GaAs}}}-1\right)x,$$

where *α*_InAs_ and *α*_GaAs_ are the lattice constants of InAs and GaAs, respectively, and *x* represents the content of indium in In*_x_*Ga_1−*x*_As. To achieve the buckling condition of the MEMS beam (i.e., *ε*_l_ = *ε*_cr_), one method is to precisely control the amount of indium; the other is to carefully vary the beam length due to the fact that *ε*_cr_ is a function of beam length, as shown in Equation (4).

**Figure 6:**
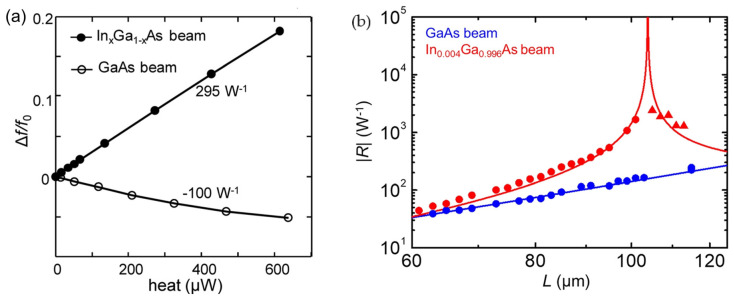
(**a**) The normalized frequency shifts are plotted as a function of the input heating power for the GaAs beam (open circles) and the In_*x*_Ga_1−*x*_As beam (full circles). Reprinted from [88], with the permission of AIP Publishing. (**b**) The absolute value of the thermal responsivities of the In_0.004_Ga_0.996_As and GaAs beams is plotted as a function of the beam length. Dots: experiment; line: theory. The In_0.004_Ga_0.996_As beams longer than 105 μm are buckled, and the polarity of their responsivities is inverted (red triangles). Reprinted from [107], with the permission of AIP Publishing.

Figure 6a shows the normalized frequency shifts of the In*_x_*Ga_1−*x*_As and unstrained GaAs MEMS bolometers as a function of heating power, which both have a geometry of 120 (length) × 30 (width) × 1.2 (thickness) μm^3^. The indium content, *x*, in the In*_x_*Ga_1−*x*_As sample is 0.001. As seen, in comparison to the unstrained GaAs resonator, the resonance frequency of the In*_x_*Ga_1−*x*_As resonator shifts to the higher frequency side from the very beginning as heating power increases, indicating the In*_x_*Ga_1−*x*_As resonator is initially buckled. Under such an initial buckling condition, the In*_x_*Ga_1−*x*_As resonator shows a 3 times larger frequency responsivity (295 W^−1^) than that of the GaAs resonator (100 W^−1^), demonstrating that the introduction of buckling conditions is useful for achieving higher thermal sensitivity for MEMS bolometers.

By varying the beam length, the MEMS beam can be modulated near the critical buckling point, achieving a larger enhancement in its thermal responsiveness. First, an In*_x_*Ga_1−*x*_As beam layer with a fixed indium concentration, *x* = 0.004, is grown, and then the unstrained GaAs and strained In_0.004_Ga_0.996_As MEMS beam resonators with various beam lengths, *L* (61–115 μm), are fabricated. Figure 6b shows the absolute value of responsivity, *R*, as a function of *L* for the GaAs and In_0.004_Ga_0.996_As beams (dots), together with theoretical expectations (solid line). As seen in Figure 6b, the In_0.004_Ga_0.996_As beams exhibit a much higher *R* than the GaAs beams with the same *L*. When the In_0.004_Ga_0.996_As MEMS beam achieves its buckling condition at *L* = 105 μm, it shows a *R* of 2400 W^−1^, which is 16 times higher than that of the GaAs sample of the same length (150 W^−1^). This result demonstrates that the introduction of a carefully designed compressive strain is useful for achieving high thermal responses for MEMS bolometers without deteriorating their detection speeds. Furthermore, the measured *R* is smaller than that of the theoretical expectation (red curve) due to a small initial deflection in the In_0.004_Ga_0.996_As MEMS beams. Consequently, a further increase in *R* is expected if the beam deflection can be suppressed.

### 4.2. Control of Mechanical Nonlinearity in MEMS Bolometer

In Section 3.1, we have mentioned that noise is a crucial factor in determining the sensitivity of MEMS bolometers, which can be greatly suppressed by applying a large driving voltage to achieve a large oscillation amplitude [90,99]. In sensing applications with MEMS bolometers, a large linear oscillation amplitude is preferable to reduce the frequency noise and improve the SNR. However, with increasing oscillation amplitude, the MEMS resonators commonly enter the nonlinear oscillation region because of the mechanical nonlinearity, where hysteretic oscillations and increased frequency noise have been observed [114,115]. This limits the potential for achieving large linear amplitudes by increasing the driving voltage. The control of mechanical nonlinearity is therefore desirable for achieving low-noise operation of MEMS resonators.

**Figure 7:**
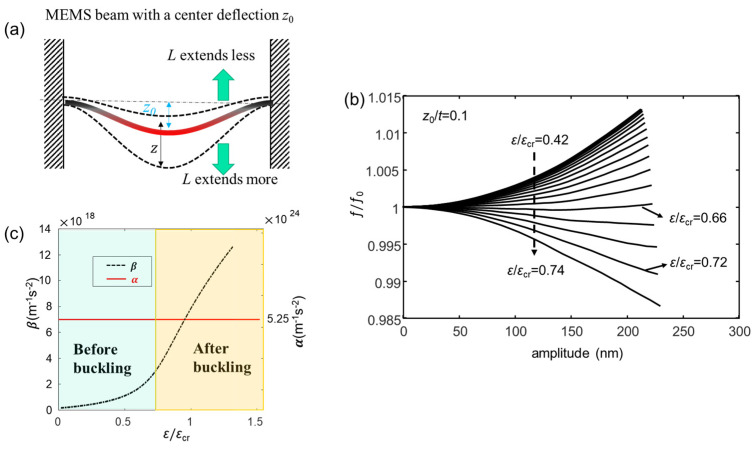
(**a**) Schematic diagram of a MEMS beam with a center deflection *z*_0_ in the steady state, which induces a quadratic softening nonlinearity in oscillation. Adapted with permission from [108]. Copyright 2023 American Physical Society. (**b**) The calculated resonance frequency (ƒ/ƒ_0_) as a function of oscillation amplitude at various *ε*/*ε*_cr_ values; the initial center deflection is *z*_0_/*t* = 0.1. The frequency is normalized by the natural frequency ƒ_0_ without oscillation. Reprinted with permission from [108]. Copyright 2023 American Physical Society. (**c**) The calculated α and *β* as a function of the compressive strain (*ε*/*ε*_cr_) at *z*_0_/*t*= 0.1.

The most evident effect of nonlinearity is the so-called geometric nonlinearity [116,117,118], which arises from the geometry of the resonator itself and its boundary conditions. The doubly clamped MEMS/NEMS resonators have the most common structures that are affected by geometric nonlinearity [119,120,121,122]. In this type of nonlinearity, the vibration dynamics of a simple harmonic resonator change into the well-known Duffing equation, which is commonly utilized for studying the nonlinear resonance behavior of MEMS resonators [116,123,124,125]. Thus, the Duffing motion equation of a flat, doubly clamped MEMS beam is expressed as [126]:

(11) $$\ddot{z}+\left[\frac{EI}{\rho S}\frac{\int {\left(\phi^{uu}\right)}^{2}du}{\int \phi^{2}du}+\frac{T_{0}}{\rho S}\frac{\int {\left(\phi^{u}\right)}^{2}du}{\phi^{2}\int du}\right]z+\left[\frac{E}{2\rho L}\frac{{\left(\int {\left(\phi^{u}\right)}^{2}du\right)}^{2}}{\int \phi^{2}du}\right]z^{3}=0,$$

where *z*(t) is the central displacement of the beam; *u* is the coordinate along the length of the beam; *ϕ*(𝑢) is the mode shape function ($\phi^{u}=\frac{\partial \phi }{\partial u}$, and $\phi^{uu}=\frac{\partial^{2}\phi }{\partial u^{2}}$); *ρ* is the density of the beam; *E* is the Young’s modulus; *L* is the beam length; *S* and *I* denote the cross-section area and the moment of inertia (*S = bt* and *I = bt^3^/*12 for beams of rectangular cross-sections, with *b* and *t* being the width and thickness of the MEMS beam, respectively); *T*_0_ is the inherent tension (positive for a tensile force and negative for a compressive force). Note that, here, the first bending mode of the MEMS beam is considered to study the mechanical nonlinearity. As seen in Equation (11), the cubic nonlinearity coefficient $\alpha =\frac{E}{2\rho L}\frac{{\left(\int {\left(\phi^{u}\right)}^{2}du\right)}^{2}}{\int \phi^{2}du}>0$ gives a hardening nonlinearity [127] and causes the resonance frequency to shift to the higher frequency side during the oscillation. However, once the symmetry of the MEMS beam is broken, e.g., if an initial center deflection exists in the MEMS beam, another quadratic (softening) nonlinearity will arise during the oscillation. As shown in Figure 7a, when the MEMS beam has an initial center deflection of *z*_0_ in the steady state, it has a new equilibrium position (*z*_0_) for the oscillation. Thus, the change in beam length caused by oscillation must be calculated from this new equilibrium position. Since the MEMS beam has a steady deflection downward, the beam extends more (hardening) in the case of downward deflection but extends less (softening) when it deflects upward in the oscillation, giving a decrease in total nonlinearity.

The motion equation of the MEMS beam with a center deflection, *z*_T_, is expressed as [108]:

(12) $$\begin{array}{c} \ddot{z}+\left[\frac{EI}{\rho S}\frac{\int {\left(\phi^{uu}\right)}^{2}du}{\int \phi^{2}du}+\frac{T_{0}}{\rho S}\frac{\int {\left(\phi^{u}\right)}^{2}du}{\phi^{2}\int du}+z_{T}^{2}\frac{E}{\rho L}\frac{{\left(\int {\left(\phi^{u}\right)}^{2}du\right)}^{2}}{\int \phi^{2}du}\right]z+\\ \left[\frac{E}{2\rho L}\frac{{\left(\int {\left(\phi^{u}\right)}^{2}du\right)}^{2}}{\int \phi^{2}du}\right]z^{3}+\left[\frac{3z_{T}E}{2\rho L}\frac{{\left(\int {\left(\phi^{u}\right)}^{2}du\right)}^{2}}{\int \phi^{2}du}\right]z^{2}=0, \end{array}$$

where *z*_T_ results from the compressive strain (*ε*) applied to the beam (*z*_0_ → *z*_T_). Compared with Equation (11), an additional quadratic nonlinear term $\beta =\frac{3z_{T}E}{2\rho L}\frac{{\left(\int {\left(\phi^{u}\right)}^{2}du\right)}^{2}}{\int \phi^{2}du}=3z_{T}\alpha$ arises, which compensates for the cubic hardening nonlinearity and leads to the suppression of the total nonlinearity [127]. Since *β* is proportional to *z*_T_ and *z*_T_ originates from the applied strain, the nonlinearity can be suppressed by precisely controlling the applied strain.

Utilizing the derived Equation (12), a numerical analysis is performed for the MEMS beam resonators with dimensions of 100 μm (*L*) × 30 μm (*b*) × 1 μm (*h*) and with *E* = 85.9 GPa and *ρ =* 5307 kg/m^3^ applied for the Young’s modulus and density of GaAs material. Figure 7b shows the calculated resonance frequency (ƒ/ƒ0) as a function of oscillation amplitude at various *ε*/*ε*_cr_ values for *z*_0_/*t* = 0.1. As seen, when the compressive strain approaches the buckling condition (*ε*/*ε*_cr_ = 0.72), the positive frequency shift reduces significantly and the frequency shift reaches a minimum at *ε*/*ε*_cr_ = 0.66, which is because of the steep increase in *β* as shown in Figure 7c. This result shows that the nonlinearity of the MEMS beam can be well suppressed by approaching the buckling condition of the MEMS beam.

Based on the above simulation result, two approaches have been experimentally demonstrated for controlling the nonlinearity. One is achieved by using thermal tuning [109], and the other is achieved by applying a preloaded lattice-mismatch strain in the MEMS beam [108].

**Figure 8:**
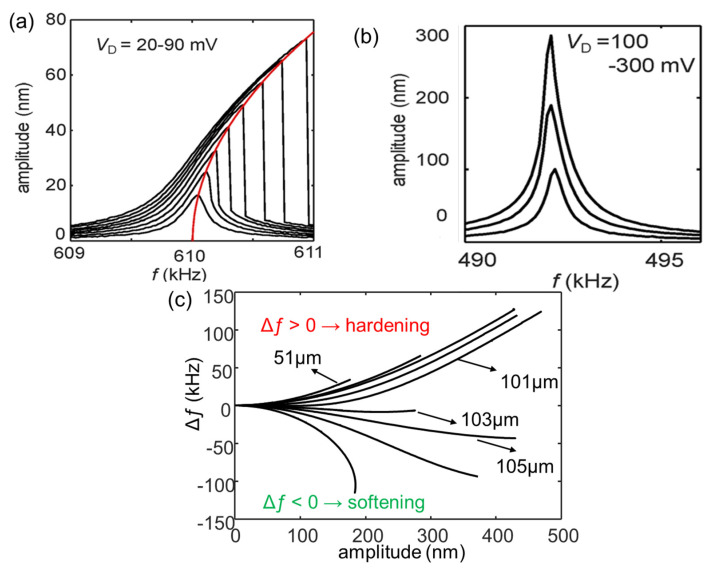
(**a**) The measured resonance spectra of the sample without thermal tuning at various driving voltages (*V*_D_ = 20–90 mV). The red curve shows that the resonance frequency shifts toward the higher frequency with increasing oscillation amplitude. Reprinted from [109], with the permission of AIP Publishing. (**b**) The measured resonance spectra of the MEMS beam with thermal tuning. Reprinted from [109], with the permission of AIP Publishing. (**c**) The measured resonance frequency shifts (Δƒ) of In_0.004_Ga_0.996_As samples with various *L*. Adapted with permission from [108]. Copyright 2023 American Physical Society.

In the case of thermal tuning, Figure 8a shows the measured resonance spectra of a MEMS resonator at various driving voltages (20–90 mV). As seen, when the driving voltage exceeds 30 mV, the oscillation enters the nonlinear regime, i.e., the resonance frequency increases with the increasing oscillation amplitude, as indicated by the red curve in Figure 8a. The linear oscillation amplitude is 30 nm for the MEMS resonator without thermal tuning. However, by using thermal tuning (i.e., applying a heating power, *P*_in_, to generate a thermal strain in the MEMS beam), the MEMS beam exhibits a ~300 nm linear oscillation amplitude in the case of *P*_in_ = 8.4 mW (buckling condition: *P*_in_ ≈ 10 mW), as shown in Figure 8b, which is 10 times larger than that of the MEMS beam without thermal tuning.

In the case of using lattice-mismatch strain, the same as what we have described in Section 4.1, the buckling condition is achieved by adding a small amount of indium into the beam structure to form In_0.004_Ga_0.996_As beams and then varying beam lengths (*L* = 51–111 μm). Figure 8c plots the measured resonance frequency shift (Δƒ) of In_0.004_Ga_0.996_As samples as a function of the oscillation amplitude with various *L*. As seen, the Δƒ changes from positive to negative as the beam length increases and reaches a minimum at *L* = 103 μm, giving a ~200 nm linear oscillation amplitude. Compared with that of the GaAs sample with the same beam length, the linear region is enhanced ~20 times. This result demonstrates the effectiveness of using lattice mismatch for controlling the mechanical nonlinearity of MEMS resonators.

### 4.3. Use of the Internal Mode Coupling Effect

The internal mode coupling effect in MEMS resonators has garnered significant attention due to its ability to substantially alter the properties of MEMS resonators. This effect arises from the internal resonance between a lower vibrational mode and a higher-order mode under the condition that their frequencies satisfy an integer ratio of 1:N, where N is the nonlinear order of the MEMS resonator [128,129,130,131,132,133]. The internal mode coupling effect enables energy transfer between two coupled vibration modes; thus, it has been proposed for the realization of frequency stabilization [114], synchronization [134], vibrational energy harvesting [135], energy dissipation control [136,137], and the detection of higher resonance modes [138]. Since it offers rich features in terms of the oscillation amplitude and frequency of MEMS resonators, it is also very attractive for sensing applications. A significant enhancement in the thermal responsivity of MEMS resonators was found when the 3:1 internal mode coupling effect was introduced [110].

**Figure 9:**
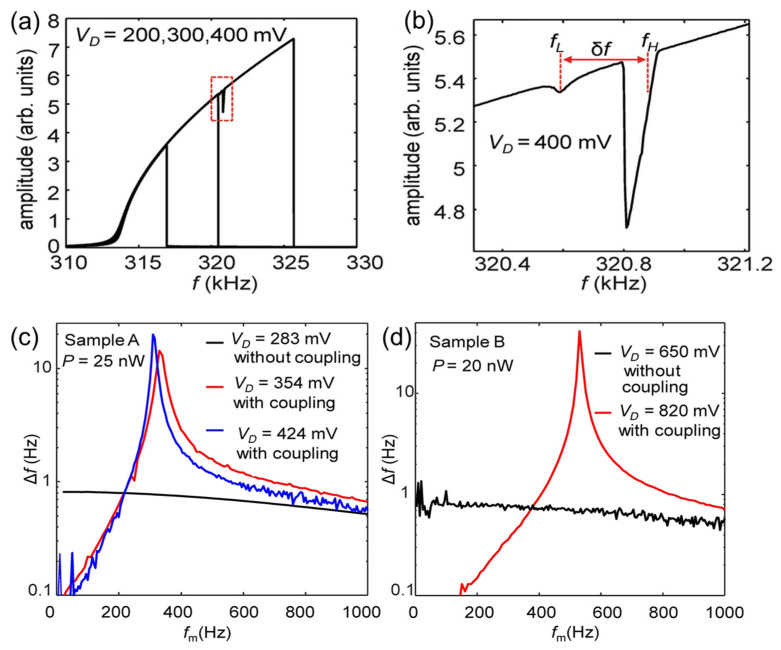
(**a**) The measured resonance spectra at *V*_D_ = 200, 300, and 400 mV. Reprinted with permission from [110]. Copyright 2020 American Physical Society. (**b**) A blow-up of the spectrum in the mode-coupling region marked by a dotted rectangle in (**a**). Reprinted with permission from [110]. Copyright 2020 American Physical Society. (**c**) Thermally induced frequency shift, Δ*f*, for sample A as a function of heat modulation frequency, *f*_m_, measured at *p* = 25 nW. The black, blue, and red curves plot the results when *V*_D_ = 283, 354, and 424 mV, respectively. Reprinted with permission from [110]. Copyright 2020 American Physical Society. (**d**) Thermally induced frequency shift, Δ*f*, for sample B as a function of heat modulation frequency, *f*_m_, measured at *p* = 20 nW. The black and red curves plot the results when *V*_D_ = 650 and 820 mV, respectively. Reprinted with permission from [110]. Copyright 2020 American Physical Society.

The designed MEMS beam resonator used for exploring 3:1 internal mode coupling has a geometry of 128 × 30 × 1.2 μm^3^, giving a fundamental bending mode (*f*_b_) with the resonance frequency of 313.8 kHz and a fundamental torsional mode (*f*_t_) with the resonance frequency of 958.0 kHz. Although *f*_b_ is slightly off by a third of *f*_t_, the larger frequency shift of *f*_b_ in the nonlinear region enables us to achieve an integer ratio equal to 3 between *f*_t_ and *f*_b_. Figure 9a shows the measured resonance spectrum of *f*_b_ at *V*_D_ = 200, 300, and 400 mV. When *f*_b_ is increased to about 320 kHz, a small reduction in the resonance amplitude is observed in the spectrum, indicating that the vibrational energy of *f*_b_ decreases under this condition. Figure 9b shows a blow-up of the spectrum in the mode-coupling region marked by the dotted red rectangle in Figure 9a. As seen, two clear drops in the amplitude appear at approximately 320.6 kHz and approximately 320.9 kHz, suggesting that internal mode coupling is formed between *f*_b_ and *f*_t_. Once the two modes couple with each other, they are renormalized into two new eigenmodes, *f*_L_ (320.6 kHz) and *f*_H_ (320.9 kHz). The energy is transferred from *f*_b_ to *f*_t_ at these two new frequencies (*f*_L_ and *f*_H_), causing two drops with a frequency difference (*δ*_f_) of 300 Hz in the resonance spectrum of the *f*_b_ mode, as shown in Figure 9b.

With this 3:1 mode coupling effect, a small amount of heating power (*p* = 25 nW) with various heat modulation frequencies, *f*_m_, is applied to the MEMS beam. Figure 9c,d shows the measured frequency shift (Δ*f*) of samples A and B as a function of *f*_m_ at various driving voltages. As seen, when the *f*_m_ reaches about 300 Hz under the internal-mode-coupling condition, a huge peak in the Δ*f* is observed, as shown by the red and blue curves in Figure 9c. In comparison to the MEMS resonator that operates outside the mode-coupling region (*V*_D_ = 283 mV), as indicated by the black curve in Figure 9c, the Δ*f* in the mode-coupling region is, respectively, 17.5 (*V*_D_ = 354 mV) and 25.0 (*V*_D_ = 424 mV) times higher. For the sample B with a *δ*_f_ *=* 500 Hz, the Δ*f* is even enhanced by a factor of 60 with the internal mode coupling effect, as shown in Figure 9d. Using this sample B, the thermal responsivity and NEP of the MEMS bolometer are further estimated by applying a heating power of 2 to 10 nW. When sample B is operated in the mode-coupling region, it exhibits a responsivity of 10,000 W^−1^, which is almost 2 orders of magnitude higher than that of being operated outside the mode-coupling region. The corresponding NEP = 23 pW/Hz^1/2^ is improved by a factor 6~7, which is found to be very close to the theoretical limits due to the thermal fluctuation noise of the MEMS beam.

### 4.4. Responsivity Enhancement Using Nanometer-Scale Hole Array Structures

In general, it is challenging to realize high thermal responsiveness and high detection speed in MEMS bolometers simultaneously. This is because both the thermal time constant (*τ*_th_ = *C*_th_/*G*_th_) and responsivity are inversely proportional to the thermal conductance (*G*_th_) of the MEMS bolometer. Longer MEMS beams offer a larger thermal response but also result in a longer response time. Thus, a route to circumvent the trade-off between detection speed and responsiveness is desirable for achieving highly sensitive sensing without deteriorating detection speed. Nano-porous structures, such as slabs with one-dimensional (1D) [139] or two-dimensional (2D) [140,141] hole arrays, have been proposed to engineer the thermal properties of materials, which is useful for improving thermal responsiveness while maintaining a fast detection speed [112]. With the hole array structure, the cross section of the beam is decreased, leading to a reduction in the thermal conductance of the MEMS beam and thus enabling it to improve thermal responsiveness. On the other hand, the material volume is also decreased by using the hole array structure, which results in a reduction in the heat capacitance of the beam, *C*_th_. Therefore, the increase in *τ*_th_ due to the decrease in *G*_th_ is partly compensated by the reduction in *C*_th_, resulting in maintaining a fast detection speed.

**Figure 10:**
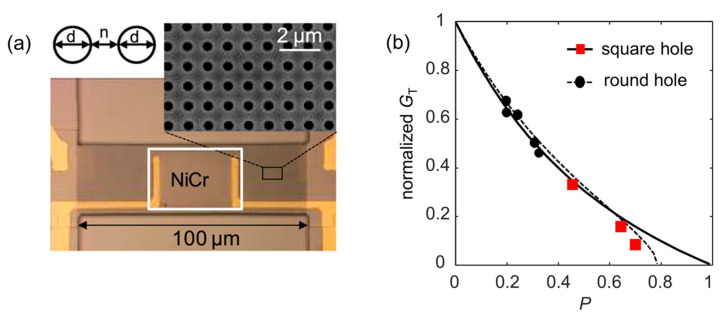
(**a**) Microscope image of a fabricated GaAs MEMS beam resonator (100 × 30 × 0.6 μm^3^) with a 2D hole array structure of a hole diameter *d* = 500 nm and a neck size *n* = 500 nm. Reprinted with permission from [112]. Copyright 2019 Zhang et al. (**b**) The normalized thermal conductance of the porous nano-mesh MEMS beams. The calculated thermal conductance of the round hole and the mesh hole is shown by the dotted line and the solid line, respectively. The square red plots are nano-mesh porous MEMS with 0.44, 0.64, and 0.69 porosities, respectively. The black dots show the thermal conductance at small porosities with a round-hole array structure. Reprinted from [111], with the permission of Yamamoto et al.

Figure 10a shows a microscope image of a fabricated MEMS beam resonator (100 × 30 × 0.6 μm^3^) with a 2D hole array structure. The inset of Figure 10a shows a blow-up of an SEM image of the hole array structure, allowing for a clear view of the homogeneous hole array. Two types of nanohole array structures, i.e., round hole array [112] and nano-mesh [111], have been fabricated on the MEMS beam. The thermal conductance of the MEMS beam has been measured to investigate how thermal sensitivity can be improved by porous structures. The red square dots in Figure 10b plot the normalized thermal conductance of the porous nano-mesh MEMS beams. For comparison, the thermal conductance at small porosities with a round-hole array is plotted as the black solid dots in Figure 10b. As seen, the thermal conductance is greatly reduced as the porosity increases. When the porosity increases to ~0.69, the thermal conductance of the porous nano-mesh beam has been reduced by over 90%. Since the thermal sensitivity is inversely proportional to the thermal conductance, an enhancement in the thermal sensitivity by over 10 times can be expected for high-porosity MEMS beams, whereas the detection speed is reduced by a factor of 3, giving an overall improvement in detection performance.

Furthermore, it has been found that the porous nanostructure will not affect the THz absorption coefficient as long as the THz wavelength is significantly larger than the featured size of the porous nanostructure. This condition is easily satisfied due to the typical THz wavelengths falling in the range of tens to hundreds of micrometers, while the porous structure exhibits nanometer-scale features.

### 4.5. Improving the Responsivity Spectrum of MEMS Bolometers

**Figure 11:**
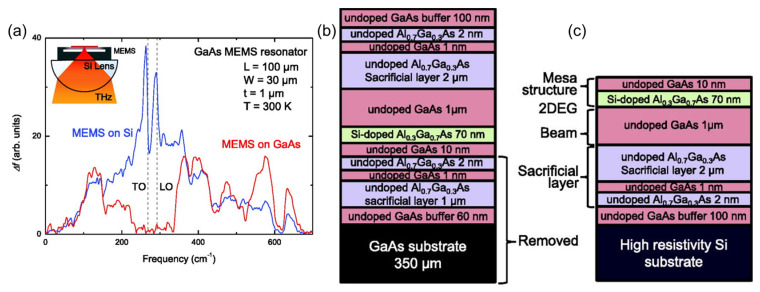
(**a**) Output Δ*f*-spectra of the MEMS bolometers fabricated on a high-resistivity Si substrate (blue) and on a GaAs substrate (red). Vertical dashed lines indicate the TO and LO frequencies in GaAs. The inset shows a schematic illustration of a MEMS bolometer mounted on a Si lens. Reprinted from [113], with the permission of AIP Publishing. (**b**) The wafer structure grown for the wafer bonding process. Reprinted from [113], with the permission of AIP Publishing. (**c**) Wafer-bonded structure used to fabricate the GaAs-based MEMS THz bolometer on a Si substrate. Reprinted from [113], with the permission of AIP Publishing.

The GaAs MEMS bolometers [59,107,112,113,142] utilize the piezoelectric property of the beam material (a III-V compound semiconductor) to drive the beam and detect oscillations. However, optical phonons in polar III–V compound semiconductors exhibit a strong interaction with THz radiation, leading to significant absorption and reflection effects [143]. This phenomenon negatively affects the response spectra of the MEMS bolometers. Figure 11a shows the output Δ*f*-spectra of the MEMS bolometers fabricated on a high-resistivity Si substrate (blue) and on a GaAs substrate (red). The red spectrum represents the signal obtained when illuminating a GaAs-based MEMS bolometer with THz electromagnetic waves from a blackbody radiation source commonly used in Fourier spectrometers. It is evident that the sensitivity spectrum is reduced by 50% in the frequency range from 160 to 240 cm^−1^ and almost vanishes in the frequency range of 240–330 cm^−1^. This behavior arises from resonant absorption at the TO phonon frequency and the transition to a negative dielectric constant within the Reststrahlenband region (from the TO phonon to the LO phonon frequency), resulting in strong reflection of electromagnetic waves.

The Si substrate has a flat transmission in the THz frequency range due to its high resistivity, which is suitable for realizing broadband THz responses. A wafer-bonding technique for fabricating a GaAs-based MEMS THz bolometer on a high-resistivity Si substrate is demonstrated [113]. Specifically, first, the original GaAs-based wafer with a reversed layer structure is grown by molecular beam epitaxy (MBE) as shown in Figure 11b, then a high-resistivity Si substrate coated with Adhesive oxide is brought into contact with the topmost layer (100 nanometer-undoped GaAs buffer) of the original wafer and bonded together. Subsequently, the bottom layers from the 350-micrometer GaAs substrate to the 2-nanometer undoped Al_0.7_Ga_0.3_As are removed using a nonselective etching. The resulting wafer-bonded structure is shown in Figure 11c. The Δ*f*-spectrum of the GaAs-based MEMS THz bolometer fabricated on a high-resistivity Si substrate is plotted by a blue curve in Figure 11a. Remarkably, the reduction or loss of sensitivity in the acoustic and optical phonon regions is no longer present, indicating a significant improvement in sensitivity and demonstrating the effectiveness of this wafer-bonding technique. Furthermore, a distinct feature is the presence of two sharp peaks near the TO and LO frequencies, which originate from an interplay between the strong reflection in the Reststrahlen band and the strong absorption at the TO phonon frequency in the thin GaAs MEMS beam structure.

## 5. Conclusions and Outlook

Thermal light sensors have the remarkable feature that they can be used to detect light of any frequency as long as it can be converted to heat. This feature is very different from that of photoconductors or rectifiers, which have limited useful frequency ranges. The very broad sensitivity of thermal sensors makes them ideal for spectroscopic applications. Indeed, the MEMS bolometers discussed in this paper have a thin metallic film light absorber, such as a NiCr thin film, which has a broad absorption spectrum covering the THz to near-IR regions.

Furthermore, the MEMS bolometers fabricated using semiconductor microfabrication technology not only have high sensitivities but also very short thermal time constants, taking advantage of the small thermal capacity of the MEMS beam. This short thermal time constant realizes excellent high-speed performance on the order of 10 kHz, which is faster by 100~1000 times than conventional pyroelectric detectors. Further speed-up is possible by reducing the size of the MEMS beam resonator. In addition, we would like to point out that the MEMS bolometer has a high degree of freedom in structure, and the sensitivity spectrum can be designed, for example, by fabricating metamaterials or other wavelength-selective structures on the surface of the MEMS beams. Such a feature is suitable for THz imaging applications using a monochromatic THz light source.

A current issue with the MEMS bolometers is their small photosensitive areas. Typical MEMS beams are designed to have dimensions in the order of ~100 μm, considering the wavelength of THz electromagnetic waves. However, this active area is too small for the conventional THz/IR light sources using ceramic heaters, whose typical size is in the order of several millimeters, as used in standard Fourier transform infrared spectrometers (FTIRs). This problem of efficient light focusing on the MEMS bolometers requires novel reducing optics. Another choice is that we completely change the light source for the FTIRs to a bright point light source or a light source that can emit collimated light. Applications of MEMS bolometers need to go along with the development of new light sources.

Concerning future directions, we would like to point out the possibilities of using other material systems for making the MEMS resonators. Thus far, we have utilized the piezoelectric properties of compound semiconductors for electrical driving and read-out of the MEMS beams. This is a very convenient and important feature of MEMS bolometers. However, as we discussed in Section 4.5, compound semiconductors, in general, strongly interact with electromagnetic waves in the THz range and exhibit reduced or no sensitivity in these frequency ranges. Therefore, the use of non-polar semiconductors, such as Si, is certainly a good direction to go. It would also be very interesting to try two-dimensional materials for very high-speed detection. 

The use of hybrid transduction of light-heat-mechanical vibration in MEMS resonators is an active area of research and is expected to lead to new and improved THz detectors in the future.

## Figures and Tables

**Table 1:** Performance of THz/IR detectors based on MEMS/NEMS resonators.

Physical Principle	Frequency Range	τ_th_	Responsivity	NEP	Reference
Torsional mode	IR	-	-	27 pW/Hz^1/2^	[72]
Torsional mode	IR	-	-	30 Hz/K	[76]
Torsional mode	IR	-	-	1000 ppm/K	[75]
Torsional mode	IR	556 µs	0.0168 W^−1^	-	[74]
Torsional mode	IR	-	10^−7^ rad/Hz^1/2^	-	[73]
Bending mode	2–3.5 THz	2.5 µs	1.5 × 108 pm/W	20 nW/Hz^1/2^	[77]
Bending mode	250 GHz–3 THz	55 μs	~100 W^−1^	500 pW/Hz^1/2^	[59]
Trampoline membrane	140 GHz	25 ms	-	100 pW/Hz^1/2^	[57]
Trampoline membrane	Light	2.4 μs	-	2 pW/Hz^1/2^	[80]
Drum	5–20 µm	17 ms	343 W^−1^	320 pW/Hz^1/2^	[79]
Thin plate	IR	0.53 ms	-	47 nW/Hz^1/2^	[78]
Optical read out	2.5 THz	1.2 μs	30 pm/nW	-	[58]
Optical read out	2.6 THz	3 μs	-	16 nW/Hz^1/2^	[81]
Optical read out	3.24–3.98 THz	100 ms	24.8 µm/µW	38.2 pW/Hz^1/2^	[82]

## Data Availability

The data presented in this study are available on request from the corresponding author.
